# Structure-based virtual screening of CYP1A1 inhibitors: towards rapid tier-one assessment of potential developmental toxicants

**DOI:** 10.1007/s00204-021-03111-2

**Published:** 2021-06-28

**Authors:** Janice Jia Ni Goh, Julian Behn, Cheng-Shoong Chong, Guorui Zhong, Sebastian Maurer-Stroh, Hao Fan, Lit-Hsin Loo

**Affiliations:** 1 Innovations in Food and Chemical Safety Programme and Bioinformatics Institute, Agency for Science, Technology, and Research, Singapore, Singapore; 2 Integrative Sciences and Engineering Programme, NUS Graduate School, National University of Singapore, Singapore, Singapore; 3 Department of Biological Sciences, National University of Singapore, Singapore, Singapore; 4 Synthetic Biology Translational Research Program, School of Medicine, National University of Singapore, Singapore, Singapore; 5 Duke-NUS Medical School, Singapore, Singapore; 6 Department of Pharmacology, Yong Loo Lin School of Medicine, National University of Singapore, Singapore, Singapore

**Keywords:** CYP1A1 inhibition, Developmental toxicity, Endocrine disruptors, Docking, Allosteric, Orthosteric

## Abstract

Cytochrome P450 1A1 (CYP1A1) metabolizes estrogens, melatonin, and other key endogenous signaling molecules critical for embryonic/fetal development. The enzyme has increasing expression during pregnancy, and its inhibition or knockout increases embryonic/fetal lethality and/or developmental problems. Here, we present a virtual screening model for CYP1A1 inhibitors based on the orthosteric and predicted allosteric sites of the enzyme. Using 1001 reference compounds with CYP1A1 activity data, we optimized the decision thresholds of our model and classified the training compounds with 68.3% balanced accuracy (91.0% sensitivity and 45.7% specificity). We applied our final model to 11 known CYP1A1 orthosteric binders and related compounds, and found that our ranking of the known orthosteric binders generally agrees with the relative activity of CYP1A1 in metabolizing these compounds. We also applied the model to 22 new test compounds with unknown/unclear CYP1A1 inhibitory activity, and predicted 16 of them are CYP1A1 inhibitors. The CYP1A1 potency and modes of inhibition of these 22 compounds were experimentally determined. We confirmed that most predicted inhibitors, including drugs contraindicated during pregnancy (amiodarone, bicalutamide, cyproterone acetate, ketoconazole, and tamoxifen) and environmental agents suspected to be endocrine disruptors (bisphenol A, diethyl and dibutyl phthalates, and zearalenone), are indeed potent inhibitors of CYP1A1. Our results suggest that virtual screening may be used as a rapid tier-one method to screen for potential CYP1A1 inhibitors, and flag them out for further experimental evaluations.

**Supplementary Information:**

The online version contains supplementary material available at 10.1007/s00204-021-03111-2.

## Introduction

Cytochrome P450 1A1 (CYP1A1) is a highly conserved enzyme that metabolizes many xenobiotics and endogenous signaling molecules (Santes-Palacios et al. 2016). The enzyme is structurally very similar to CYP1A2, another member from the same CYP family; but the tissue distributions of these two enzymes are quite different. CYP1A1 is highly expressed in the adult lungs, liver, gastrointestinal tract, skin, and other tissues (Nishimura et al. 2003; Choudhary et al. 2005); but CYP1A2 is mostly expressed in the liver (Nishimura et al. 2003; Choudhary et al. 2005). Furthermore, CYP1A1, but not CYP1A2, can be detected in the placenta, embryo, and fetus (Omiecinski et al. 1990; Hakkola et al. 1996; Nishimura et al. 2003; Choudhary et al. 2003, 2005). The enzyme is constitutively active in certain embryonic tissues within specific time windows during the early development of mouse (Choudhary et al. 2003; Campbell et al. 2005) and zebra fish (Otte et al. 2010), as early as the gastrulation stage (Otte et al. 2010). It has high catalytic activity for the oxidative metabolism (including 2-, 4-, 15α- and 16α-hydroxylations) of female sex hormones, estradiol (E_2_) and estrone (E_1_) (Lee et al. 2003). Hydroxylated estrogen metabolites, such as 15α-hydroxyestriol (also called estetrol or E_4_) and 16α-hydroxyestradiol (also called estriol or E_3_), are produced by human fetus (Gurpide et al. 1966) and increase substantially during pregnancy (Adlercreutz and Martin 1976; Holinka et al. 2008). Besides estrogens, CYP1A1 also metabolizes melatonin (Ma et al. 2005), and arachidonic acid (AA) and eicosapentaenoic acid (Schwarz et al. 2004), which are essential for normal embryonic and/or fetal development (Tamura et al. 2008; Yanes et al. 2010). Therefore, it is not surprising that previous animal studies have found that exogenous CYP1A1 inhibitors disrupt embryonic development (Wassenberg and Di Giulio 2004; Yin et al. 2014); and CYP1 gene knockouts increase the rates of embryonic lethality and birth defects (Dragin et al. 2008), and decrease the weights of key organs and the body of fetuses that manage to develop into adults (Dragin et al. 2008; Agbor et al. 2012). All of these results show that functioning CYP1A1 is required for normal embryonic and/or fetal development. Therefore, exogenous inhibitors of CYP1A1, including drugs or environmental agents, may disrupt important endocrine signaling processes, and lead to unintended developmental toxicity effects.

**Fig. 1:**
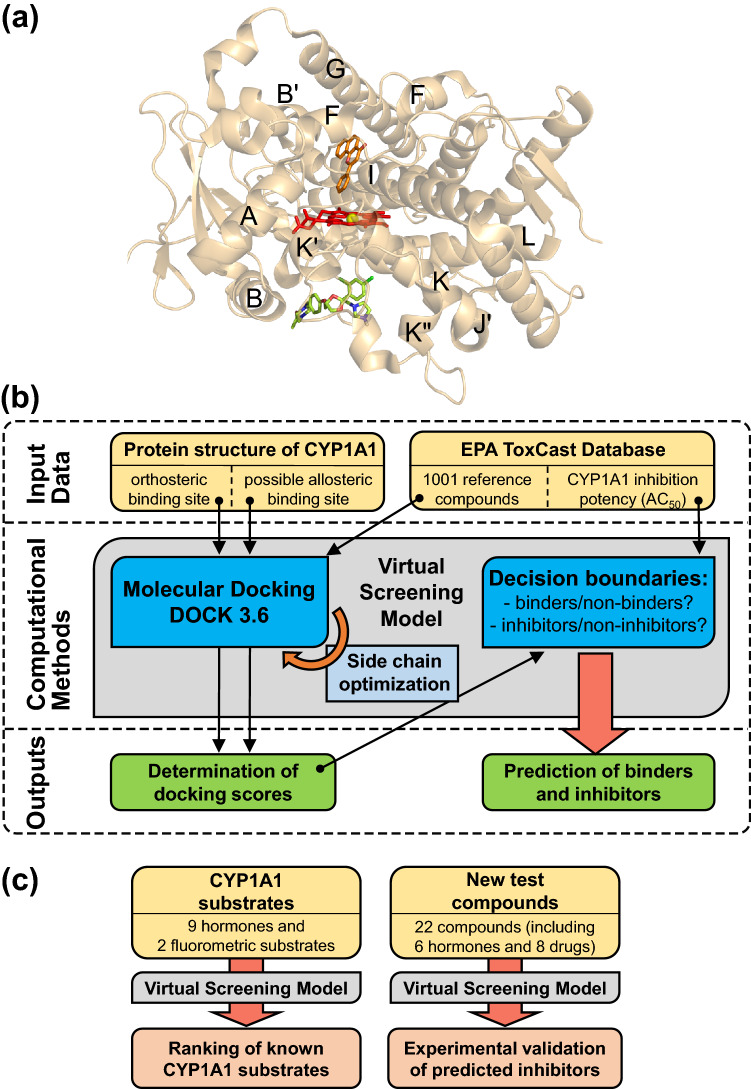
Building a virtual screening model for CYP1A1 inhibitors based on the orthosteric and allosteric sites of the enzyme**. a** Overview of the previously reported orthosteric binding site and the predicted allosteric binding site on human CYP1A1 (black letters = helices names (Walsh et al. 2013), red sticks = heme, orange sticks = co-crystallized ligand α-naphthoflavone, green sticks = docking pose of ketoconazole in the allosteric site; protein structure is based on PDB ID: 4I8V). **b** Flowchart showing the overall computational procedure to train and use the virtual screening model. **c** Three sets of test compounds unused during the training process were used to validate the virtual screening model (color figure online)

CYP1A1 catalyzes the oxidative metabolisms of specific substrates. The heme enzyme has a narrow and planar active site that restricts substrate orientation (Walsh et al. 2013). The heme forms the “floor” of the pocket (Fig. 1a). Several residues, such as Phe 224, that were previously found to be important for the recognition and binding of substrates provide constraints to the pocket (Walsh et al. 2013). Therefore, xenobiotics that bind to the active site may compete with other endogenous substrates, such as estrogens or melatonin, and inhibit the metabolism of these substrates. X-ray crystallography of CYP1A1 and several potent inhibitors, including α-naphthoflavone, bergamottin, and erlotinib, have found that these inhibitors bind to the active site (Walsh et al. 2013; Bart and Scott 2018). These crystal structures enable the use of computational methods, such as docking analysis, to rapidly screen for molecules that may bind to the active site. Several previous studies have built virtual screening models based on the active site to identify CYP1A1 inhibitors as candidates for anti-cancer drugs (Sridhar et al. 2012; Joshi et al. 2017).

However, the activity of a CYP may also be modulated through allosteric sites away from the active site (Hardy and Wells 2004). There are very few previous reports of allosteric sites that can inhibit the activity of a CYP. It has been suggested that a CYP interacts with cytochrome P450 oxidoreductase (POR) at its proximal side, where the heme group of the CYP is closest to its surface (Bridges et al. 1998), to facilitate electron transfer from NADPH to the CYP active site and oxidization of the bound substrate (Denisov et al. 2005). For efficient electron transfer, stable complex formation and correct positioning of these proteins at the contact regions are crucial (Hlavica et al. 2003). Electrostatic interactions between CYP and POR were postulated to be responsible for their binding (Mayuzumi et al. 1993). In particular, basic residues on the proximal CYP surface were found to be important for direct interaction or correct geometric positioning with acidic residues on POR (Im and Waskell 2011). More recently, it has been proposed that surface exposed hydrophobic residues may also play a vital role in the interaction (Kenaan et al. 2011). Notably, the structure of a self-sufficient bacterial CYP enzyme (Sevrioukova et al. 1999) containing both heme and flavine mononucleotide (FMN) domains revealed that the latter is positioned at the proximal surface of the heme domain. Since the FMN domain can also be found in human POR, similar interactions may occur in the human CYP–POR complex. Although there are more than 50 CYPs present in the human genome, there is only one gene encoding POR (Kandel and Lampe 2014). Thus, CYP1A1 is likely to interact with POR via similar mechanisms. Previous studies have found that POR mutations can cause disordered steroidogenesis in human (Huang et al. 2005), and affect the activities of many CYP enzymes, including CYP1A1 (Hayashi et al. 2003) and 1A2 (Agrawal et al. 2008). Furthermore, similar to CYP1A1, POR knockout in mice results in embryonic lethality and multiple developmental defects (Shen et al. 2002). Thus, the inclusion of additional allosteric sites for CYP1A1 located at or near potential CYP–POR binding sites may help us to more accurately predict CYP1A1 inhibitors.

Here, we present a virtual screening model for CYP1A1 inhibitors based on the orthosteric and a predicted allosteric site of CYP1A1 that overlaps with residues that may be critical for the interaction between CYP and POR. Our strategy was to use docking analysis (Fan et al. 2009; Jaladanki et al. 2021) to estimate the binding affinities via “docking scores” of a large set of reference compounds to the two sites, systematically identify possible relationships between the docking scores and the changes in CYP1A1 activity induced by these compounds, and derive docking-score thresholds that may optimally separate between binders vs non-binders, in particular between inhibitors vs non-inhibitors (Fig. 1b). We hypothesize that our virtual screening model can be used to computationally screen for CYP1A1 inhibitors, which may cause embryonic and/or fetal developmental toxicities. To test this hypothesis, we applied our final model to 11 known CYP1A1 orthosteric binders and related compounds, and compared the docking scores of these compounds to the known activity levels of CYP1A1 in metabolizing these compounds. We further tested the model by applying it to 22 new compounds with unknown/unclear CYP1A1 activity, many of which are drugs known to induce fetal development toxicities in humans (Fig. 1c). We then experimentally validated the potency and modes of inhibition of those compounds predicted to be CYP1A1 inhibitors.

## Results

### Identification of potential allosteric CYP1A1 binding sites

Our virtual screening model is based on a previously published structure (PDB ID: 4I8V) (Walsh et al. 2013) of CYP1A1 and α-naphthoflavone, which binds to the orthosteric site (Supplementary Material 1—Table S1) surrounded by helices *F*, *G* and *I* and the loops around the *B'* region (Fig. 1a). To identify potential allosteric sites based on the structure, we used a computational program—“metaPocket 2.0” that predicts potential binding sites on the protein surface based on a consensus of eight geometry- and energy-based computational methods (Zhang et al. 2011); and another program—“CryptoSite” that predicts cryptic binding sites that may become apparent only after a conformational change of the protein (Cimermancic et al. 2016). CryptoSite computes a per-residue-score that indicates the chance of a residue being part of a cryptic site, and residues with scores larger than 10 are more likely to be part of such sites.

**Fig. 2:**
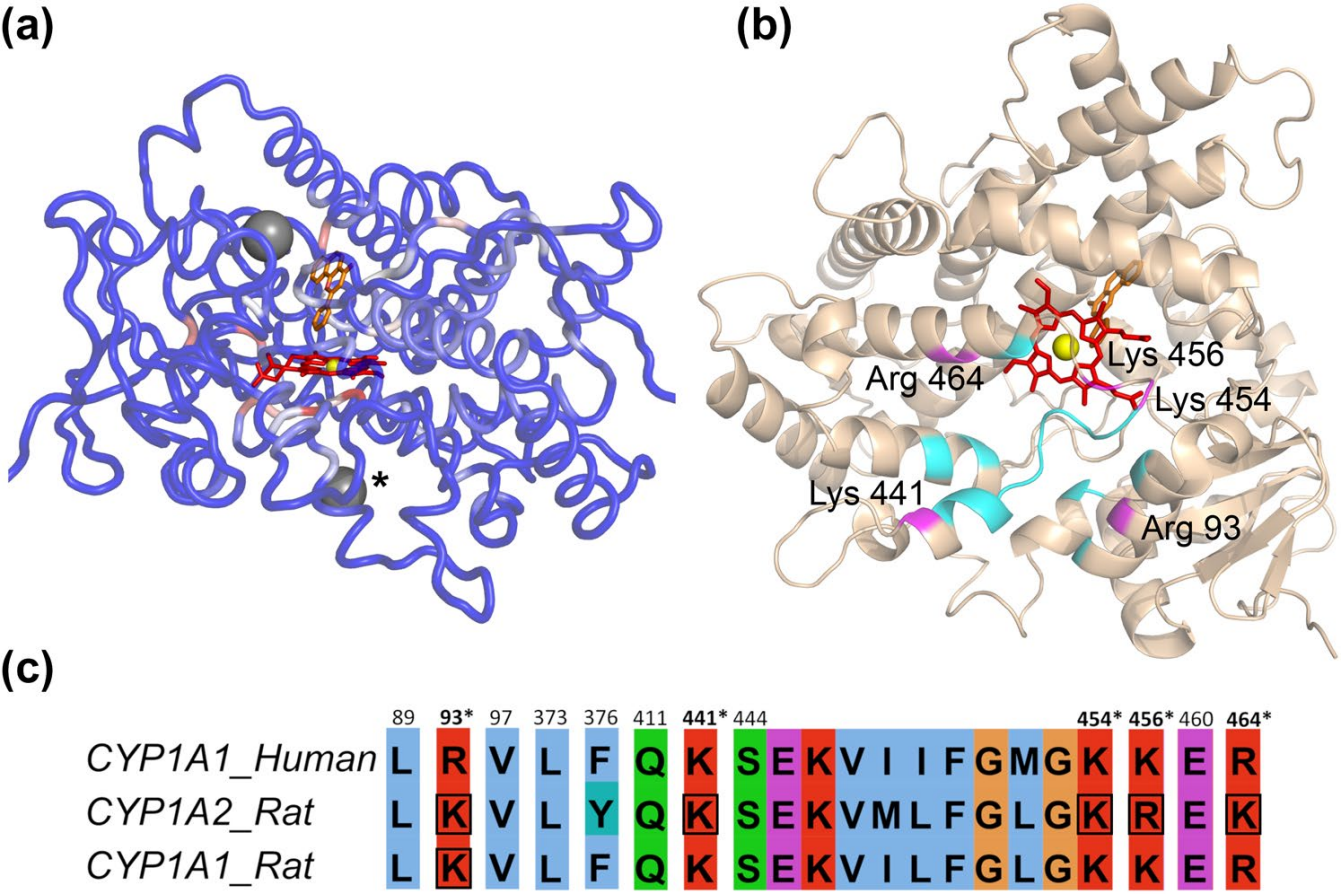
Predicting a novel CYP1A1 allosteric site at the CYP-POR interface. **a** Visualization of both “metaPocket 2.0” and “CryptoSite” outputs on human CYP1A1 (grey spheres = predicted metaPocket clusters not located at the orthosteric site; * = the final selected allosteric site; red sticks = heme; orange sticks = the co-crystallized ligand, α-naphthoflavone). The backbone is colored according to the CryptoSite scores with a cutoff at 10 (blue = minimum, white = middle, red = maximum score values). **b** Structural model of the proximal surface of human CYP1A1. The predicted allosteric site is centered at the meander region of the enzyme (in cyan) and overlaps (in magenta) with five labelled basic POR-binding residues. **c** Multiple sequence alignment of mammalian CYP1 enzymes with the 21 residues making up the predicted allosteric binding site (numbers above the alignment = residue positions with respect to human CYP1A1, bolded and asterisked positions = overlaps of the residues with possible POR-binding residues, boxes = POR binding residues that have been experimentally verified in the other mammalian CYP1 enzymes)(color figure online)

Four clusters of residues were predicted and ranked by metaPocket as potential binding sites. Two of them are located at the known orthosteric site, and thus not further considered. The third cluster is located on the protein surface on the “proximal” side of the heme (opposite to the active site) (Fig. 2a). In this cluster, 68.8% of the residues have CryptoSite scores above the threshold. The fourth cluster, which has a lower rank than the third cluster, is located on the protein surface above the active site. However, only 22.8% of the residues in this cluster have CryptoSite scores above the threshold (Fig. 2a). Therefore, we selected the third cluster as a candidate of an allosteric site (Supplementary Material 1—Table S2). The site is surrounded by helices *B*, *J'*, *K* and *L* and the loop region between the *K''* and *L* helices (Fig. 1a).

### Binders to the allosteric site may disrupt CYP–POR interaction

We found that the potential allosteric site is located at the proximal surface of CYP1A1 with most of the predicted binding residues concentrated at the meander coil of the enzyme (cyan regions in Fig. 2b). To examine the possibility that there may be an overlap between the allosteric and POR binding sites, we performed a literature search and identified 17 reported POR-binding residues in the CYP1A family proteins in rats and rabbits (Supplementary Material 1—Table S3). By performing a multi-species sequence alignment (Methods), we mapped the identified residues to the surface of human CYP1A1 and then compared them to the allosteric site. Of the 21 residues defining the allosteric site (Supplementary Material 1—Table S2), we found five basic residues that overlapped with the mapped POR-binding residues on CYP1A1 (Fig. 2c). Three of them (Lys 441, Lys 454 and Lys 456) are located at the meander coil region between helices K′ and L, while the other two residues (Arg 93 and Arg 464) are, respectively, positioned in helices B and L (Fig. 2b). Other mapped POR-binding residues in CYP1A1 are far (more than 10 Å) away from the allosteric site, except for Arg 98 that is completely solvent-exposed and partially blocked by Arg 93 from the allosteric site.

Site-directed mutagenesis and chemical modifications of homologous basic residues in mammalian CYP1A1 and CYP1A2 have shown that these five overlapping residues (Fig. 2c and Supplementary Material 1—Table S3) are essential to the CYP–POR interaction. For example, covalent modification or swapping of Lys 453 (mapped to Lys 454 here) in mammalian CYP1A2 with glutamic acid was demonstrated to increase the apparent dissociation constant (*K*_d_) of the interaction and/or diminish POR-dependent catalytic activity (Furuya et al. 1989; Shimizu et al. 1991; Mayuzumi et al. 1993). Similarly, ablation of positive charge at the other four overlapping residues can also perturb CYP–POR interactions (Shimizu et al. 1991; Shen and Strobel 1992; Mayuzumi et al. 1993), underscoring the importance of these residues as contact regions for an electrostatic interaction with POR. Therefore, the overlap between POR-binding residues and the allosteric site suggests that an allosteric binder may disrupt CYP1A1–POR binding in a direct manner. The high affinity (~ 5–110 nM) (Kandel and Lampe 2014) and small contact area (~ 967 Å^2^) (Sevrioukova et al. 1999) of a CYP–POR interaction may make it more amenable to disruptions by small molecule inhibitors (Thompson et al. 2012). Therefore, we hypothesize that the inclusion of this site into our virtual screening model may improve the accuracy in predicting CYP1A1 inhibitors.

### Reference compounds and activity data from ToxCast

**Fig. 3:**
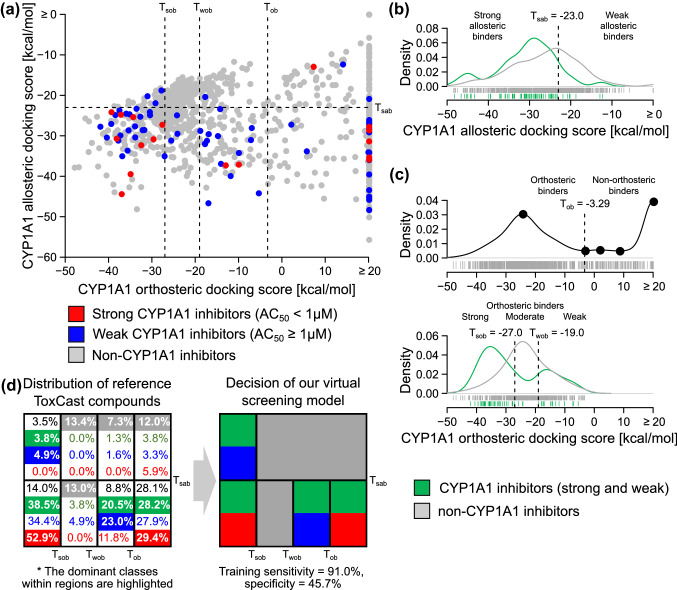
Determining decision thresholds for inhibitors based on ToxCast compounds. **a** Scatter plot showing the allosteric and orthosteric docking scores for the 1001 reference compounds from ToxCast (blue or red dots = weak or strong CYP1A1 inhibitors based on ToxCast activity data, respectively; gray dots = non-CYP1A1 inhibitors; *T*_sob_ or *T*_wob_ = strong or weak orthosteric binder threshold, respectively; *T*_ob_ = orthosteric binder threshold; *T*_sab_ = strong allosteric binder threshold). **b** Density plot showing the probability distribution functions of the allosteric docking scores for CYP1A1 inhibitors (green) or non-inhibitors (gray). **c** Density plots showing the probability distribution functions of the orthosteric docking scores for all compounds (black), CYP1A1 inhibitors (green), or non-inhibitors (gray). **d** Distributions of the reference compounds at the eight regions (left grid map) defined by the four docking-score thresholds (*T*_sob_, *T*_wob_, *T*_ob_, and *T*_sab_). The dominant classes within the regions are highlighted (green = CYP1A1 inhibitors, blue = weak CYP1A1 inhibitors, red = strong CYP1A1 inhibitors, gray = non-CYP1A1 inhibitors), and used to derive the final decision rule of our virtual screening model (right grid map) (color figure online)

To develop a virtual screening model for CYP1A1 inhibitors, we used established docking protocols (Fan et al. 2009; Jaladanki et al. 2021) (Methods) to estimate the binding affinities via “docking scores” of a large set of reference compounds to the two sites. We used the US EPA’s ToxCast database (InVitroDB v2.0) (Kavlock et al. 2012), which provides bioactivity data for > 2000 chemical compounds, including a high-throughput CYP1A1 activity assay based on a fluorometric substrate, resorufin benzyl ether (BzRes). After performing quality control and removing compounds that overlap with our validation compounds or are not suitable for docking analysis (Methods), we ended up with 1001 reference compounds (78 are CYP1A1 inhibitors, and 923 are non-CYP1A1 inhibitors). Among the inhibitors, 17 of them have “activity concentration at 50% of maximal activity” (AC_50_) < 1 µM, and these compounds are called “strong” inhibitors. The other 61 inhibitors with 1 ≤ AC_50_ < 100 µM are called “weak” inhibitors. For some of the compounds, the docking algorithm could not determine any valid docking geometry and all poses got “bumped” by the orthosteric site. For these cases, we applied an unfavorable docking score of + ∞ to the respective compounds. Furthermore, if there was more than one valid docking pose for a compound, we only considered the pose with the best docking score. The results were a set of CYP1A1 orthosteric and allosteric docking scores for the 1001 reference compounds (Fig. 3a and Supplementary Material 2).

The large set of reference compounds allow us to systematically identify possible relationships between the CYP1A1 docking scores and inhibition potency, and derive decision thresholds that may optimally separate inhibitors from non-inhibitors. We preferred to use simple linear thresholds, so that the classification of the compounds can be easily interpreted. The intended application of our virtual screening model is to perform tier-one in silico screens for drug-candidate or environmental-agent safety assessments. Further experimental tests will always be performed to confirm the “hits” and detect false positives misidentified during the in silico screens. We expect that the percentage of inhibitors in the tested compounds is usually low. For example, only 7.79% of our reference compounds are inhibitors. Therefore, we prefer a virtual screening model with higher sensitivity than specificity. For this study, we always searched for decision thresholds that provide the maximum sensitivity while maintaining a balanced accuracy at or higher than the 95th percentile level among all possible thresholds.

### Threshold for allosteric binders

When studying the distribution of the allosteric docking scores, we found that none of the 1001 tested compounds is being bumped from the allosteric site (Fig. 3b). The result suggests that the site is promiscuous and most compounds may bind to the site but to different extents. Since the site is closed to the potential CYP–POR interacting site, these binders may inhibit the activity of CYP1A1. However, there are other mechanisms to inhibit CYP1A1 (such as binding to the orthosteric site), thus an inhibitor may not necessarily be an allosteric binder. Interestingly, we found that inhibitors in the reference compounds generally have more favorable (negative) allosteric docking scores than non-inhibitors (Fig. 3b). The results suggest that predicted stronger allosteric-site binders are more likely to inhibit CYP1A1 catalytic activity than weaker allosteric-site binders. By performing a grid search across all the allosteric docking scores (Methods), we found that the 95th percentile of all achievable balanced accuracy values was 63.4%. Among all the thresholds with balanced accuracy at or higher than this level, we further found an optimum threshold for strong allosteric binders (*T*_sab_ = −23.0 kcal/mol, Fig. 3b) that maximizes the sensitivity (91.0%). The balanced accuracy and specificity of this threshold are 63.6% and 36.2%, respectively.

### Thresholds for orthosteric binders

However, we observed a very different trend for the distribution of the orthosteric docking scores. We found that 282 (28.2%) of the reference compounds have docking scores larger than 20 kcal/mol, 127 of them are bumped (Fig. 3c). The results suggest that the orthosteric site is more selective than the allosteric site, likely due to the narrow and planar structure of the orthosteric site (Introduction). We note that an orthosteric binder may be an inhibitor and/or a substrate of CYP1A1, but a substrate may not necessarily have a strong potency in inhibiting the activity of CYP1A1. Therefore, unlike the allosteric docking scores, we cannot use the CYP1A1 activity level to separate binders vs non-binders. Instead, we determined the inflection points along the docking score distribution (Fig. 3c, upper panel), and used the first local minima after the first local maxima as a threshold for orthosteric binders (*T*_ob_ = −3.29 kcal/mol). We assume that most of the compounds with docking scores lower than *T*_ob_ are binders to the orthosteric site.

Surprisingly, when studying the docking-score distributions of inhibitors and non-inhibitors predicted to be orthosteric binders, we found a “gap” in the distribution of inhibitors (Fig. 3c, lower panel). Most of the inhibitors have either highly or lowly negative docking scores, but very few inhibitors have moderately negative docking scores. Importantly, there are also many non-inhibitors with docking scores within this gap, which is around the peak of the orthosteric score distribution for all orthosteric binders (Fig. 3c, upper panel). Therefore, two thresholds are required to optimally separate the inhibitors and non-inhibitors. As before, by performing a grid search across the orthosteric docking scores (Methods), we found that the 95th-percentile balanced accuracy is 66.4%. We further identified two optimum thresholds for strong orthosteric binders (*T*_sob_ = −27.0 kcal/mol) and weak orthosteric binders (*T*_wob_ = −19.0 kcal/mol) that maximize the sensitivity (94.3%) in classifying the orthosteric binders into either inhibitors or non-inhibitors. The balanced accuracy and specificity of these thresholds are 69.2% and 44.1%, respectively. When we used all the three orthosteric thresholds together and considered all the reference compounds, the performance became 64.1% sensitivity and 66.5% specificity (65.3% balanced accuracy). Therefore, a virtual screening model based on orthosteric docking scores alone would have higher specificity but lower sensitivity than a model based on allosteric docking scores alone. The lower sensitivity mostly stems from the large number of inhibitors with unfavorable orthosteric docking scores (orthosteric scores > *T*_ob_ and allosteric scores < *T*_sab_, Fig. 3a). Overall, we observed associations between the orthosteric and/or allosteric docking scores and CYP1A1 inhibition potency, which agree with our hypothesis that binders to one site or both sites may inhibit CYP1A1.

### Final virtual screening model

Using all the four thresholds, we divided all the reference compounds into eight classes of compounds with different predicted CYP1A1 orthosteric and allosteric binding profiles (Fig. 3a). Then, we estimated the distributions of all non-inhibitors, all inhibitors, only strong inhibitors, and only weak inhibitors in these classes of compounds based on the ToxCast CYP1A1 activity data (Fig. 3d, left panel). Because the total numbers of different categories of inhibitors were not the same within a class of compounds, the percentage of all inhibitors was usually not the sum of the percentages of strong and weak inhibitors. We found that the probability of finding a non-inhibitor is higher than finding an inhibitor in predicted weak allosteric binders (allosteric scores > *T*_sab_), except when the compound is also predicted to be a strong orthosteric binder (orthosteric scores < *T*_sob_). Conversely, the probability of finding an inhibitor is higher than finding a non-inhibitor in predicted strong allosteric binders (allosteric scores < *T*_sab_), except when the compound is also predicted to be a moderate orthosteric binder (*T*_sob_ < orthosteric scores < *T*_wob_). Interestingly, the strong inhibitors have the highest probabilities to be found in either the strong allosteric-only binders (allosteric scores < *T*_sab_ and orthosteric scores > *T*_ob_) or the strong allosteric and orthosteric binders (allosteric scores < *T*_sab_ and orthosteric scores < *T*_sob_). Based on these observations, we derived a final rule to classify a compound as CYP1A1 inhibitors or non-inhibitors according to its allosteric and orthosteric docking scores (Fig. 3d, right panel). The performance of the final rule on the 1001 reference compounds was 91.0% sensitivity and 45.7% specificity (68.4% balanced accuracy).

### Application to known binders and other related compounds

**Fig. 4:**
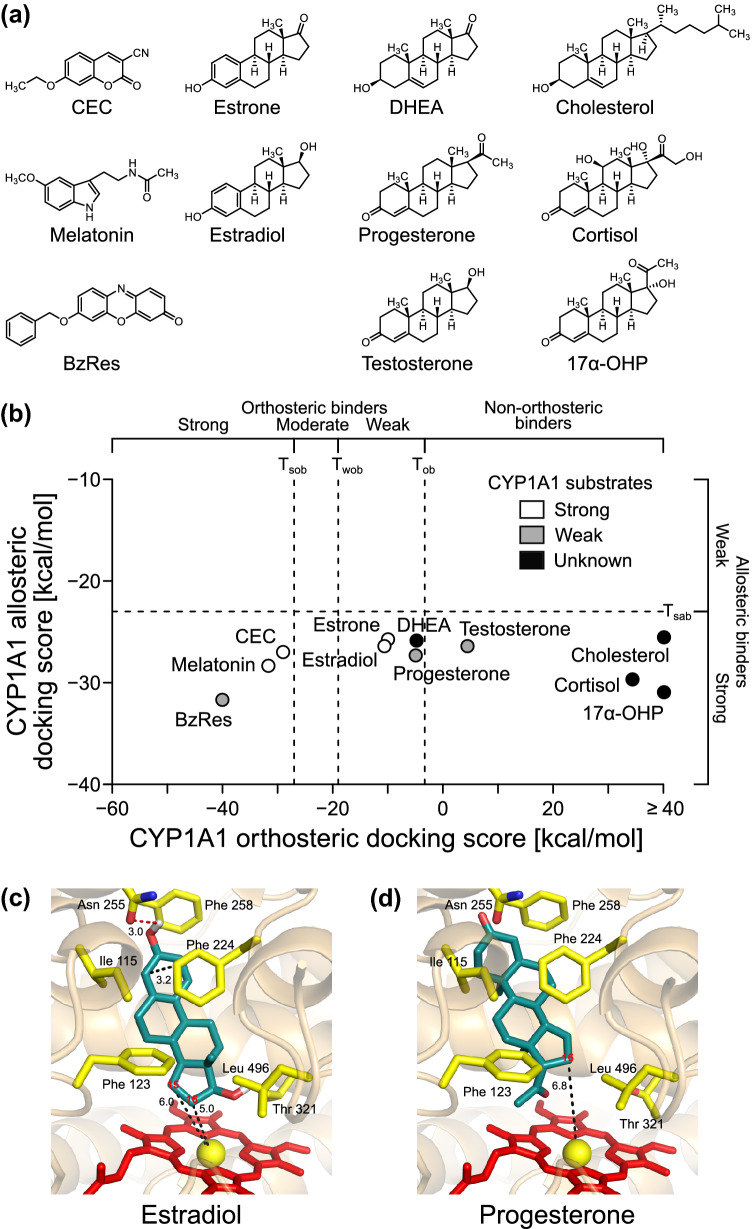
Validating the model with known CYP1A1 substrates and other related compounds. **a** Structures of the 11 known CYP1A1 substrates and other related compounds (CEC = 3-cyano-7-ethoxycoumarin, BzRes = resorufin benzyl ether, DHEA = dehydroepiandrosterone, 17α-OHP = 17α-hydroxyprogesterone). **b** Scatter plot showing the allosteric and orthosteric docking scores for the compounds (white dots = strong CYP1A1 substrates, gray dots = weak CYP1A1 substrates, black dots = unknown compounds, please refer to the Results section for the definitions of these categories; *T*_sob_ or *T*_wob_ = strong or weak orthosteric binder threshold, respectively; *T*_ob_ = orthosteric binder threshold; *T*_sab_ = strong allosteric binder threshold). Docking poses of **c** estradiol or **d** progesterone at the CYP1A1 orthosteric site (red sticks = heme; yellow sticks = selected sidechains; turquoise sticks = estradiol or progesterone; black dashed lines = distances in Å; red numbers = key sites of metabolism). For progesterone, the refined pose is shown (color figure online)

To verify that our virtual screening model can properly recognize orthosteric binders of CYP1A1, we applied the final model without any further modification to 11 known CYP1A1 substrates and related compounds, which include nine hormones and two fluorometric substrates (Fig. 4a). Based on experimental measurements from other previous studies, we categorized the compounds into three categories: “strong” CYP1A1 substrates (known to be hydroxylated in at least one reaction with metabolic activity > 1 nmol/min/nmol CYP1A1), “weak” CYP1A1 substrates (known to be hydroxylated in at least one reaction between 0.1 and 1 nmol/min/nmol CYP1A1), and “unknown” compounds (not known to be hydroxylated, or no hydroxylation in the checked reactions beyond the sensitivity limits of the assays used).

The nine hormones were melatonin, estrone (E_1_), estradiol (E_2_), dehydroepiandrosterone (DHEA), testosterone, progesterone, cortisol, 17α-hydroxyprogesterone (17α-OHP), and cholesterol (Fig. 4a). A previous study of melatonin metabolism by human CYPs found that this hormone is a strong substrate of CYP1A1 (Ma et al. 2005). A recent review of steroid hormones hydroxylated by human CYPs (Niwa et al. 2015) found that E_1_ and E_2_ are strong substrates of CYP1A1; testosterone and progesterone are weak substrates of CYP1A1; and DHEA, cortisol, 17α-OHP, and cholesterol are known substrates of other CYPs. The review did not provide usable information about CYP1A1’s activity on the last group of hormones, suggesting that either no CYP1A1 study has been performed on these hormones or they may not be the main substrates of CYP1A1. DHEA is known to inhibit CYP1A1 (Klinger et al. 2002), and thus may be a CYP1A1 substrate. Despite of the lack of information, this group of hormones were still included in our analysis, because they are structurally related to E_2_ (Fig. 4a).

We docked these nine hormones to the orthosteric and allosteric sites of CYP1A1 (Fig. 4b and Supplementary Material 3). All of them were not part of the reference compounds, and thus they were not used to derive the thresholds. We found that all the three strong CYP1A1 substrates (melatonin, E_2_, and E_1_) have the best orthosteric dockings scores (−31.79, −10.73, and −10.08 kcal/mol, respectively), which are all below the orthosteric binder threshold (*T*_ob_ = −3.29 kcal/mol). Our model correctly predicted that melatonin, E_2_, and E_1_ are orthosteric binders. The docking pose of E_2_ is characterized by an aromatic π–π-interaction between Phe 224 and the steroidal ring A moiety at ~ 3.2 Å distance (Fig. 4c). Furthermore, the key C15 and C16 positions are the closest to the center of the heme group, with C–Fe distances of 6.0 and 5.0 Å, respectively. E_1_ also has a very similar docking pose (data not shown). The results are in strong agreement with the expected hydroxylation at these key positions (Introduction). Progesterone and DHEA were the other two hormones predicted to be weak orthosteric binders, but their orthosteric docking scores (−5.05 and −4.90 kcal/mol, respectively) are very close to *T*_ob_ (Fig. 4b). The docking pose of progesterone (Fig. 4d) agrees with a previous finding that CYP1A1 catalyzes the 16α-hydroxylation of the hormone (Schwarz et al. 2000).

Among all the steroid hormones, E_2_, E_1_, progesterone, and DHEA have the lowest orthosteric docking scores, and these four hormones are predicted to be weak orthosteric binders of CYP1A1 (Fig. 4b). Previous studies have found that these four hormones may inhibit CYP1A1 (Eugster et al. 1993; Klinger et al. 2002), but their relative potency levels are unclear. Thus, we decided to include these hormones as part of our test compounds, whose ability to inhibit CYP1A1 were later experimentally determined and compared. All the other steroid hormones have docking scores > *T*_ob_, including another weak CYP1A1 substrate, testosterone. Most of these hormones have very similar allosteric docking scores (Fig. 4b); therefore, the difference in their activity is likely due to the difference in their binding affinity to the orthosteric site.

We also docked two fluorometric substrates of CYP1A1, namely, 3-cyano-7-ethoxycoumarin (CEC) and resorufin benzyl ether (BzRes). The ToxCast program (Kavlock et al. 2012) used BzRes to measure CYP1A1 activity, and we used the resulting data to optimize the thresholds for our virtual screening model (Fig. 3). However, a previous comparison of the activities of 29 rat and human CYPs on nine fluorometric substrates found that CEC is a much stronger substrate for human CYP1A1 than BzRes (40.2 and 0.457 pmol/min/pmol CYP1A1, respectively) (Stresser et al. 2002). Using our model, we found that the docking scores of these two substrates are −29.1 and −40.1 kcal/mol (for orthosteric site), and −26.9 and −31.6 kcal/mol (for allosteric site), respectively (Fig. 4b). Therefore, our model correctly predicted that both compounds are strong orthosteric binders and inhibitors of CYP1A1 (< *T*_sob_ = −27.0 kcal/mol). However, CEC has an allosteric docking score much closer to the allosteric binder threshold (*T*_sab_ = −23.0 kcal/mol), and thus is less likely to bind to the allosteric site compared to BzRes. To avoid potential interference with the allosteric site and also due to the stronger reported metabolic activity of the substrate (Stresser et al. 2002), we decided to use CEC, but not BzRes, as the fluorometric CYP1A1 substrate for our validation experiments.

### Application to new test compounds

We applied our model to a new set of 18 test compounds completely unused during the threshold optimization process. They include two steroid hormones (E_3_ and E_4_), eight known or potential endocrine disruptors (anastrozole, bisphenol A or BPA, cyproterone acetate, diethyl phthalate, dibutyl phthalate, propyl paraben, tamoxifen, and zearalenone), and eight known lung toxicants from a previous study of pulmonary toxicity (Lee et al. 2018) (amiodarone, bicalutamide, cyclophosphamide, 2,4′-DDT, diacetyl, ketoconazole, β-myrcene, and *p*-phenylenediamine). The lung toxicants were included, because CYP1A1 is also highly expressed in the adult lung tissues (Nishimura et al. 2003), and thus these compounds may interact with the enzyme. Together with the four steroid hormone substrates predicted to be weak CYP1A1 inhibitors earlier, these 22 test compounds provide a rich and diverse set of molecules to validate our virtual screening model.

Nine of the test compounds are FDA-approved drugs, namely, amiodarone, anastrozole, bicalutamide, cyclophosphamide, cyproterone acetate, E_2_, ketoconazole, progesterone, and tamoxifen. Thus, the human fetal developmental effects of most of these drugs have been clinically evaluated, and we obtained their assigned FDA pregnancy categories from their packaging labels (Table 1 and Methods). Two of them (bicalutamide and E_2_) are of Category X, which have studies in animals or humans showing fetal abnormalities and the risk of using these drugs in a pregnant woman clearly outweighs any possible benefit. Five of them (amiodarone, anastrozole, cyclophosphamide, cyproterone acetate, and tamoxifen) are of Category D, which have positive evidence of human fetal risk based on adverse reaction data from investigational or marketing experience or studies in humans, but potential benefits may warrant the use of these drugs in pregnant women despite of the risks. Finally, ketoconazole is of Category C, which cause some adverse fetal effects on animal reproduction studies, but there is no adequate or well-controlled human study to confirm these effects; and progesterone is of Category B, which have low risks to fetus based on animal studies. Thus, most of these nine drugs are known development toxicants to humans, except for ketoconazole and progesterone, whose human developmental effects are unclear/unstudied.

**Table 1:** Potency and mode of inhibition of the test compounds

Name	CASN	FDA pregnancy category	IC_50_	(95%CI)	*K*_i_	(95%CI)	α	(95% CI)	MOI
Ketoconazole	65277-42-1	C	0.099	(0.066–0.13)	0.11	(0.063–0.24)	0.68	(0.27–1.4)	Noncompetitive
Zearalenone	17924-92-4	–	0.7	(0.49–0.91)	0.66	(0.43–1.1)	3.5	(1.5–8.3)	Mixed
Bisphenol A	80-05-7	–	2.4	(2–2.8)	0.53	(0.36–0.85)	9.1	(3.8–40)	Competitive
Amiodarone	19774-82-4	D	7.8	(4.3–11)	1.4	(0.85–2.7)	4.7	(1.7–15)	Competitive
Cyproterone acetate	427-51-0	D	9.9	(1.5–18)	8.8	(5.7–15)	7	(2.8–28)	Competitive
Dibutyl phthalate	84-74-2	–	10	(5.6–15)	1.6	(1.2–2.4)	13	(5.5–210)	Competitive
Tamoxifen	10540-29-1	D	12	(7.2–16)	3.6	(2–8.2)	7.2	(2.2–44)	Competitive
Bicalutamide	90357-06-5	X	14	(11–18)	6.6	(4.4–11)	2.7	(1.2–5.9)	Mixed
Diethyl phthalate	84-66-2	–	38	(21–56)	10	(6.4–18)	8.4	(3.2–72)	Competitive
Propyl paraben	94-13-3	–	39	(29–48)	17	(11–27)	6.2	(2.5–21)	Competitive
Progesterone	57-83-0	B	41	(19–63)	20	(12–41)	4.3	(1.5–18)	Competitive
Estradiol	50-28-2	X	43	(17–70)	13	(8.2–22)	4.3	(1.7–15)	Competitive
β-Myrcene	123-35-3	–	52	(42–62)	11	(7.3–17)	3.5	(1.6–7.6)	Mixed
DHEA	53-43-0	–	130	(67–200)	–	–	–	–	–
Anastrozole	120511-73-1	D	190	(19–370)	–	–	–	–	–
Estetrol	15183-37-6	–	> 200	–	–	–	–	–	–
2,4′-DDT	789-02-6	–	> 200	–	–	–	–	–	–
Estrone	53-16-7	–	> 200	–	–	–	–	–	–
*p*-Phenylenediamine	106-50-3	–	> 200	–	–	–	–	–	–
Cyclophosphamide	6055-19-2	D	∞	–	–	–	–	–	–
Diacetyl	431-03-8	–	∞	–	–	–	–	–	–
Estriol	50-27-1	–	∞	–	–	–	–	–	–

*DHEA *dehydroepiandrosterone; *MOI* mode of inhibition; *95% CI* 95% confidence interval; unit for IC_50_ and *K*_i_ = µM; ∞ = the best fitted model was the constant model; > 200 = the estimated IC_50_ value was larger than the maximum tested concentration, 200 µM; –  not tested, computed, or available; see Methods for the rules used to assign MOI based on α)

**Fig. 5:**
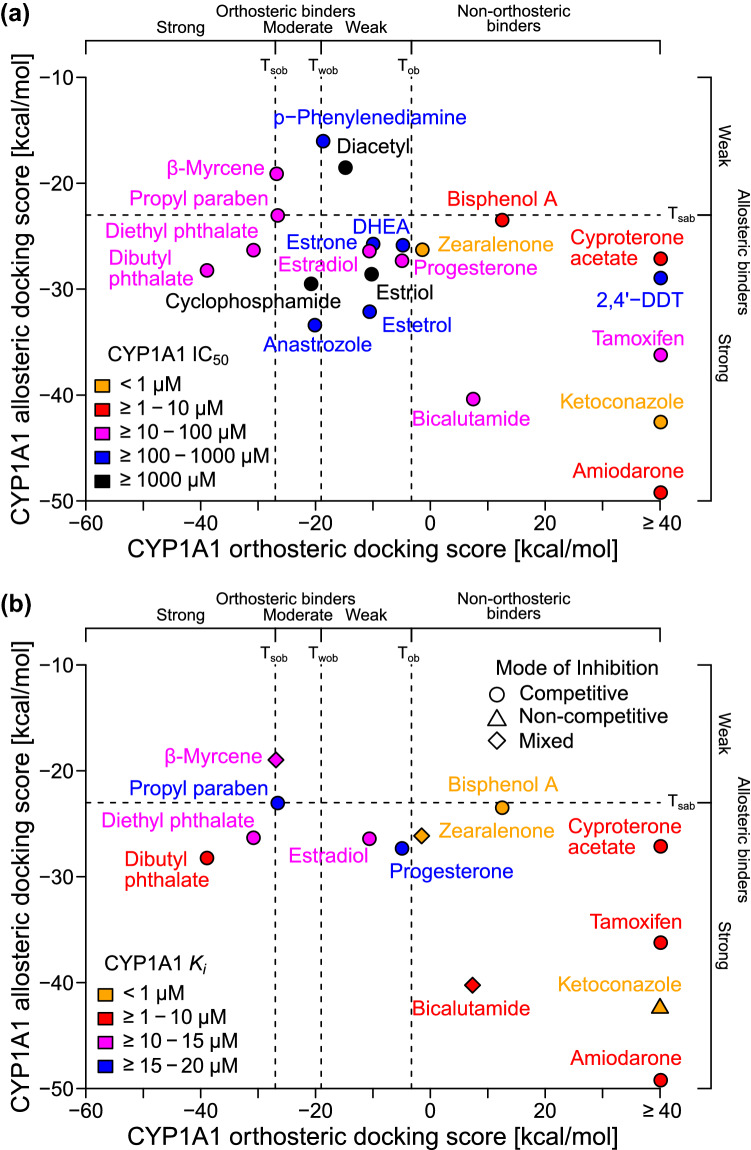
Validating the model with new test compounds using a CYP1A1 activity inhibition assay. Scatter plot showing the experimentally determined **a** IC_50_ values and **b** *K*_i_ values and predicted modes of inhibition for 22 test compounds (DHEA = dehydroepiandrosterone; *T*_sob_ or *T*_wob_ = strong or weak orthosteric binder threshold, respectively; *T*_ob_ = orthosteric binder threshold; *T*_sab_ = strong allosteric binder threshold)

Our models predicted that 16 of the test compounds are CYP1A1 inhibitors and six are non-CYP1A1 inhibitors (Fig. 5a). To validate the predictions, we experimentally determined the half maximal inhibitory concentration (IC_50_) of these compounds in inhibiting the activity of CYP1A1 in metabolizing CEC (Table 1). We found 13 compounds with IC_50_ < 100 µM (“measured inhibitors”) and 9 compounds with IC_50_ ≥ 100 µM (“measured very weak or non-inhibitors”). Our model correctly identified 11 of the 13 measured inhibitors (sensitivity = 84.6%) and 4 of the 9 measured very weak or non-inhibitors (specificity = 44.4%). The validation performance is very similar to the training performance that we obtained during the earlier model building process (Fig. 3d). The only two mis-detected inhibitors (β-myrcene and propyl paraben) are very close to the *T*_sob_ decision boundary. Furthermore, the two most potent measured inhibitors (zearalenone and ketoconazole, both with IC_50_ < 1 µM) were predicted to be allosteric-only binders (Fig. 5a). The results agree with our earlier observation from the reference compounds that 29.4% of strong CYP1A1 inhibitors are predicted to be allosteric-only binders (Fig. 3d).

Among all the tested steroid hormones, we confirmed that both E_2_ and progesterone have the highest potency, IC_50_ = 43 µM (17–70 µM, 95%CI) and 41 µM (19–63 µM, 95%CI), respectively. Four other steroid hormones (E_1_, E_3_, E_4_, and DHEA) were false positives. In earlier analysis (Fig. 4b), we found that the orthosteric docking score of DHEA is very close to the orthosteric binder threshold (*T*_ob_). We confirmed that DHEA is a very weak CYP1A1 inhibitor with IC_50_ = 130 µM (67–200 µM, 95%CI). Furthermore, among the two tested phthalates, dibutyl phthalate has both more favorable orthosteric and allosteric docking scores than diethyl phthalate (Fig. 5a), despite of the fact that dibutyl phthalate has longer chain length than diethyl phthalate. We predicted that both phthalates are strong CYP1A1 inhibitors, but dibutyl phthalate may have a higher potency than diethyl phthalate. Again, we confirmed that the IC_50_ for dibutyl and diethyl phthalates are 10 and 38 µM, respectively.

### Dissociation constants and modes of inhibition

IC_50_ is a potency measurement determined at a single substrate concentration, and thus the measurement depends on the concentration of the substrate used. To determine the potency and mode of inhibition of the compounds independent of the substrate concentration used, we performed additional experiments at multiple substrate concentrations (Methods). The resulted data allowed us to model the enzymatic kinetics and estimate the equilibrium dissociation constant for the enzyme–inhibitor complex (“*K*_*i*_”), and also the degree to which the binding of inhibitor changes the affinity of the enzyme for substrate (“α”) (Methods). When α is much larger than one, the binding of an inhibitor prevents the binding of a substrate, thus the inhibition is “competitive”; when α is close to one, the binding of an inhibitor does not change the binding of a substrate, thus the inhibition is “noncompetitive”; otherwise, the inhibition is “mixed”. We only determined *K*_*i*_ and α for the 13 CYP1A1 inhibitors with IC_50_ < 100 µM.

Again, we found that the *K*_i_ values generally agree with our model predictions (Table 1). The highly potent compounds (*K*_i_ < 10 µM) were predicted to be either strong allosteric-only binders or strong orthosteric and allosteric binders (Fig. 5b). Interestingly, ketoconazole, zearalenone, and BPA are the three compounds with the highest potency (*K*_i_ < 1 µM). We also found that most of the inhibitors induced competitive inhibition, except for ketoconazole, which is non-competitive, and β-myrcene, which is mixed (Fig. 5b). Together, our results suggest that our virtual screening model is working as expected, and correctly predicted most of the CYP1A1 inhibitors while maintaining a reasonable specificity.

## Discussion

We have developed a virtual screening model for CYP1A1 inhibitors based on the orthosteric site and a predicted allosteric site of the enzyme. Binders to the allosteric site of CYP1A1 may directly disrupt the interaction and electron transfers between POR and CYP1A1, and thus inhibit CYP1A1’s catalytic activity. However, binders to the allosteric site may also induce other long-range effects that can result in conformational changes at the enzyme’s active site (Grover 2013) or indirect interferences of the interaction between CYP1A1 and POR or other proteins (Modell et al. 2016). This may explain why we found that many compounds predicted to be allosteric-only binders induce competitive inhibition of CYP1A1 (Fig. 5b and Table 1). Regardless of the underlying mechanisms, our results suggest that a more sensitive predictor for CYP1A1 inhibition can be obtained when both the orthosteric and allosteric sites are considered (91% sensitivity) compared to when only the orthosteric site is considered (64.1% sensitivity) while maintaining similar or slightly higher levels of balanced accuracy (68.4% and 65.3%, respectively). Due to the known roles and knockout/inhibition effects of CYP1A1 to embryonic and/or fetal development (Introduction), it is very likely that CYP1A1 inhibitors may cause developmental toxicity.

In our study, we found that several drugs, including those from FDA Pregnancy Category C, D or X, are predicted to be CYP1A1 inhibitors (Fig. 5). Among them, we have experimentally verified that amiodarone, bicalutamide, ketoconazole, tamoxifen, and cyproterone acetate are potent CYP1A1 inhibitors (all with *K*_i_ < 10 μM). Amiodarone is an antiarrhythmic drug known to inhibit several human CYPs (Ohyama et al. 2000). We found that the drug has *K*_i_ = 1.4 μM (0.85–2.7 μM, 95%CI) and α = 4.7 (1.7–15, 95%CI) for CYP1A1 inhibition, suggesting that it has a mixed mode of inhibition. Our measurements were very close to the values determined by another previous study for desethylamiodarone (DEA), an active metabolite of amiodarone (*K*_i_ = 1.5 μM, α = 5.7) (Ohyama et al. 2000). Interestingly, that study compared eight CYPs, and found that DEA has the highest potency in inhibiting CYP1A1. Therefore, both amiodarone and DEA are potent CYP1A1 inhibitors. Previous studies have found that gestational exposure to amiodarone may lead to intrauterine growth restriction in rats (Hill and Reasor 1991) and humans (Widerhorn et al. 1991). Therefore, our results suggest that CYP1A1 inhibition may be one of the mechanisms underlying the observed amiodarone effects.

Another drug that we tested was ketoconazole, an antifungal drug known to inhibit CYP3A4’s orthosteric site by ligating its azole nitrogen to the enzyme’s heme group (Ekroos and Sjogren 2006). However, our model predicted that ketoconazole is a strong CYP1A1 inhibitor that only binds to CYP1A1’s allosteric site. We experimentally verified that the drug has *K*_i_ = 110 nM (63–240 nM, 95%CI) and α = 0.68 (0.27–1.4, 95%CI). Again, our measurements were very close to the values determined from a previous study of the same compound for CYP1A1 inhibition (*K*_i_ = 36.6 nM) (Paine et al. 1999). The measured α value suggests that ketoconazole is a non-competitive inhibitor of CYP1A1, which agrees with our prediction that the drug does not bind to the orthosteric site of CYP1A1. These results provide a strong support to our hypothesis that binding to the allosteric site alone is sufficient to inhibit CYP1A1 activity. Interestingly, another related antifungal drug, fluconazole, was one of our reference chemicals and predicted to be a CYP1A1 inhibitor by our model (Supplementary Material 2). A previous study found that fluconazole can inhibit CYP1A1’s metabolism of AA, and the formation of biologically active AA metabolites, such as hydroxyeicosatetraenoic acids (HETE) (El-Sherbeni and El-Kadi 2016). Other previous animal studies have found that both ketoconazole and fluconazole induce embryo lethality and teratogenicity at high doses, but similar effects have so far been observed only for fluconazole in humans (Pilmis et al. 2015). Therefore, FDA classified ketoconazole as Category C, but fluconazole as Category D. Due to their more severe developmental toxicity effects than CYP1A1 knockouts, the two azole antifungals are likely to inhibit additional targets beyond CYP1A1.

We also found that several suspected endocrine disruptors, namely, BPA, zearalenone, dibutyl phthalate (DBP), diethyl phthalate (DEP), and propyl paraben are CYP1A1 inhibitors. Phthalates and BPA are used in plastics, parabens are preservatives used in personal care products, and zearalenone is a naturally occurring mycotoxin that can be found in high concentrations in dairy products and cereals. All of them are suspected endocrine disruptors that may activate estrogen receptors under in vitro conditions (Blair et al. 2000). In previous studies, these compounds were also found to inhibit ethoxyresorufin-*O*-deethylation (EROD), a marker for CYP1A1 activity, in rat liver microsomes (Ozaki et al. 2016), human MCF-7 cell line (Yu et al. 2004), or mouse Hepa-1c1c7 cell line (Jeong et al. 2000). However, due to the existence of other CYPs and/or proteins that may regulate CYP1A1’s expression (such as the aryl hydrocarbon receptor, AhR) in the assays used by these studies, the observed effects may not be specifically attributed to direct CYP1A1 inhibition (Burke et al. 1994). In our study, we experimentally confirmed that human CYP1A1 can be inhibited by these compounds (*K*_i_ = 0.53, 0.66, 1.6, 10, and 17 μM, respectively). The high potency of BPA, zearalenone, and DBP (*K*_i_ < 2 μM) suggests that these suspected endocrine disruptors may interfere with the endocrine systems not only by directly modulating the activity of key signaling receptors (such as estrogen receptors), but also by indirectly inhibiting the activity of enzymes that generate or convert key signaling molecules (such as estrogens) in these systems.

Besides CYP1A1, several other CYPs are also highly expressed in the fetus or placenta. They include CYP1B1, CYP2C8, CYP2D6, CYP2E1, CYP3A4, CYP3A5 and CYP3A7 (Hakkola et al. 1998), which may also play important roles during embryonic or fetal development. Our approach of building a virtual screening model based on orthosteric and allosteric sites is general, and thus should be applicable to these other CYPs as well. By combining models for several CYPs and other key enzymes required for embryonic/fetal development, we may be able to more accurately predict compounds that can cause developmental toxicity, and filter out potential developmental toxicants for further experimental tests during drug-candidate or environmental-agent safety screening.

## Methods

### Topological modelling of the spatial distribution of potential POR-binding residues

To identify CYP1A1 residues important for the binding to POR, a literature search was conducted to obtain collective evidence on possible binding residues based on past studies in enzymes from the CYP1 family. A multi-species sequence alignment was then generated by MAFFT (https://mafft.cbrc.jp/alignment/server/) using parameters for the L-INS-i iterative refinement method (Katoh et al. 2002). The illustration for the multi-sequence alignment was made using Jalview 2.10.3b13 and colored according to physicochemical properties using the Clustal X scheme at http://www.jalview.org/help/html/colourSchemes/clustal.html. Protein sequences used for the alignment were obtained from the UniProt database (The UniProt Consortium 2021) and their respective accession IDs, related enzyme and POR binding residue information as well as the literature references they were derived from are summarized in Supplementary Material 1—Table S3. Based on the results of the alignment, these binding residues were then mapped to their corresponding residues on the human CYP1A1 structure used. Finally, a comparison was made between the POR-binding residues and the predicted allosteric binding site residues to identify overlapping residues.

### Reference compounds and activity data

The October 2015 release version of the “ToxCast & Tox21 Summary Files” data set was retrieved from US EPA server (ftp://newftp.epa.gov/COMPTOX/High_Throughput_Screening_Data/Previous_Data/ToxCast_Data_Release_Oct_2015/). For this study, the assay with endpoint name NVS_ADME_hCYP1A1 was used. The assay was based on the fluorescence intensity signal changes due to the metabolism of BzRes catalyzed by human CYP1A1. The reference compound set was derived from the original ToxCast data set through a multi-step scheme. First, all compounds that overlap with the test compounds used in the validation studies were removed. Then, we used the assay variables and model fitting parameters from the ToxCast data to decide if a compound is an inhibitor or not. Compounds that were found to be inactive at the single-concentration tests and thus not selected for multi-concentration tests were assigned as “non-inhibitors”. In addition, compounds tested in multiple concentrations but with a “constant” fitted concentration–response model were also assigned as “non-inhibitors”. Compounds with a non-constant fitted model, but with either AC_50_ value larger than 10 μM or maximum response values less than 10% were discarded from the reference compound set, because the activity data for these compounds was not sufficient to clearly distinguish them as either “inhibitors” or “non-inhibitors”. All the remaining compounds were assigned as “inhibitors”. Finally, compounds with missing or non-valid SMILES strings or compounds with less than 5 atoms had to be removed from the reference set due to their unsuitability for the docking approach. The final CYP1A1 reference data set consisted of 1001 compounds, of which 78 were CYP1A1 inhibitors (Supplementary Material 2).

### Protein structure

The protein structure for CYP1A1 (PDB ID: 4I8V, UniProt accession: P04798) (Walsh et al. 2013) was downloaded from the RCSB PDB (https://www.rcsb.org/). Only chain A was considered. The co-crystallized ligand α-naphthoflavone was removed from the protein structure before docking, while the cofactor heme was kept. The procedures used to identify the allosteric site are described in the Results section. Graphics for all structural models were created using Pymol 1.7.2.1.

### Docking

We used the DOCK 3.6 (Mysinger and Shoichet 2010) for virtual screening of the ToxCast reference data set against the CYP1A1 protein structure. Docking was performed at the known orthosteric binding pocket and at our predicted allosteric binding site. For each site, we performed a cycle of docking runs to improve the docking poses of the ToxCast reference compounds. Between two consecutive docking runs, we always performed a local sidechain optimization using the “Protein Local Optimization Program” (PLOP) program (Jacobson et al. 2002). For progesterone, we found some steric clashes with the rigid orthosteric pocket from the CYP1A1 protein structure. Therefore, we did small sidechain refinement to the crystal structure and re-dock the hormone. The docked compounds were ranked by the docking energy function that is the sum of van der Waals, Poisson–Boltzmann electrostatic, and ligand desolvation penalty terms. For some compounds the algorithm could not determine a valid docking geometry (in case all poses got “bumped”). In this case, we applied an unfavorable docking score of + ∞ to the bumped compound. Furthermore, if there was more than one valid docking pose for a certain compound, we only considered the best scoring pose.

### Allosteric and orthosteric thresholds

For the allosteric docking scores, we first capped the maximum scores to 0 kcal/mol, i.e., assigning 0 kcal/mol to compounds with docking scores larger than the value. Then, a grid search was used to determine the sensitivity and specificity in classifying inhibitors and non-inhibitors based on a threshold: $T_{{{\text{sab}}}} \in \left\{ { - 60, - 59,~ - 58, \ldots , - 2, - 1,~0} \right\}$. After that, all the thresholds with balanced accuracy levels equal or larger than the 95th percentile value were identified, and the final threshold was chosen to have the maximum sensitivity among the identified thresholds.

For the orthosteric docking scores, we first capped the maximum scores to 20 kcal/mol, i.e., assigning 20 kcal/mol to compounds with docking scores larger than the value. Then, we estimated the probability density function (PDF) of the scores using the density() function with smoothing bandwidth = 3 under the R environment (v.3.5.2; The R Foundation, Vienna, Austria). Inflection points were determined from the smoothed curve, and the threshold for orthosteric binders (*T*_ob_) was determined to be the first local minima after the first local maxima. Then, for all predicted orthosteric binders (orthosteric score < *T*_ob_), a grid search was used to determine the sensitivity and specificity in classifying inhibitors and non-inhibitors based on two thresholds: $T_{{{\text{sob}}}} \in \left\{ { - 60, - 59,~ - 58, \ldots , - 5,~ - 4} \right\}$ and $T_{{{\text{wob}}}} \in \left\{ { - 60, - 59,~ - 58, \ldots , - 5,~ - 4} \right\}$, where $T_{{{\text{sob}}}} < T_{{{\text{wob}}}}$. After that, all the threshold pairs with balanced accuracy levels equal or larger than the 95th percentile value were identified, and the final threshold pair was chosen to have the maximum sensitivity among the identified pairs.

### Materials

High-performance liquid chromatography (HPLC)-grade acetonitrile (ACN) was used (VWR, Pennsylvania, USA). All test compounds are of analytical-grade (≥ 99.0% purity; from Sigma-Aldrich, St. Louis, USA or Cayman Chemicals, Michigan, USA). Water was obtained using a water purification system (Milli-Q; Millipore, Billerica, MA, USA).

### CYP1A1 activity assays

The compounds were evaluated for their potency in inhibiting CYP1A1’s metabolism of CEC. The assay was based on bactosomes containing human recombinant CYP1A1 (rCYP1A1) and cytochrome P450 oxidoreductase (#EZ014; Cypex, Scotland, UK), and NADPH regenerating system containing NADPH A (NADP+ and glucose-6-phosphate) and B (glucose-6-phosphate dehydrogenase) solutions (#451220 and #451200, respectively; BD Gentest, Woburn, MA, USA). First, the test compounds were dissolved and titrated in ACN, and preincubated with a 30 µL incubation mixture consisting of 5 µM CEC, 5 pmol/mL rCYP1A1, 1:100 NADPH B, and 100 mM potassium phosphate buffer (pH 7.4) for 15 min at 37 °C. The final concentrations of the test compounds were 0.00, 0.091, 0.274, 0.823, 2.47, 7.41, 22.2, 66.7, and 200 µM; except for ketoconazole were 0.00, 0.009, 0.027, 0.082, 0.247, 0.741, 2.22, 6.67, and 20 µM. The test solutions were prepared in 384-well plates (Corning, New York, USA), and in quadruple technical replicates. Then, the reactions were initiated by adding 10 µL NADPH A to the test solutions, yielding 50 µL final test mixtures with 2% ACN (v/v). After incubation for 2.5 min at 37 °C, the reactions were quenched with 30 µL ice-cold ACN. Finally, the fluorescence intensity of the generated metabolites was immediately quantified using a multimode plate reader (Synergy Mx; BioTek, VT, USA) at λ_ex_ = 405 nm and λ_em_ = 460 nm. In every batch of experiments, ketoconazole was always included as a positive control. At least two independent experimental replicates were performed for each compound. To determine the *K*_i_ and α values of a compound, the same CYP1A1 activity assay was repeated six times in different CEC concentrations (1.56, 3.125, 6.25, 12.5, 25.0, and 50.0 µM). The final concentrations of the compounds were kept the same.

### ***IC***_***50***_*** computation***

For each experimental replicate, the median fluorescence intensity value across the four technical replicates was first determined. Then, an average activity value was obtained by taking the mean of the median values from at least two experimental replicates. For each compound, the ratios of the average activity levels at all the tested concentrations with respect to the 0 µM control were determined, and used to fit two concentration–response-curve models. The first model is a two-parameter log-logistic model, $\Delta _{{{\text{model}}}} ([I]) = 1/(1 + (b(\log ([I]) - \log ({\text{IC}}_{{50}} )))$, where [*I*] is the inhibitor concentration, *b* is the steepness of the fitted curve, and IC_50_ is the half maximal inhibitory concentration. The second model is a constant model, $\Delta _{{{\text{model}}}} ([I]) = 1$. The Akaike's Information Criterion (AIC) (Akaike 1974) was then used to identify the best fitted model. Compounds optimally fitted by the constant model were deemed to be “non-inhibitors”, and their IC_50_ values were set to ∞. The DRC library (v3.0–1) under the R environment (v.3.5.2; The R Foundation, Vienna, Austria) was used to fit the models, including the estimation of the 95% confidence intervals of the estimated IC_50_ values.

### ***K***_***i***_*** and α computation***

First, we estimated the maximum rate of the reaction (${V}_{\text{max}}$) and the Michaelis–Menten constant of the enzyme (${K}_{\mathrm{m}}$) using a non-linear regression model, $v = (V_{{\max }} \cdot [S])/(K_{{\text{m}}} + [S])$, where *v* is the velocity of the reaction and [*S*] is the concentration of the substrate. The model was fitted only with measurements obtained without the inhibitor (i.e., the concentration of the inhibitor, [*I*] = 0). Then, we estimated the inhibition constant of the inhibitor values of (${K}_{\mathrm{i}}$) and the degree to which the binding of inhibitor changes the affinity of the enzyme for substrate ($\alpha$) using another non-linear regression model, $v = {{\left( {V_{{\max }} \cdot \left[ S \right]} \right)} \mathord{\left/ {\vphantom {{\left( {V_{{\max }} \cdot \left[ S \right]} \right)} {\left\{ {K_{{\text{m}}} \cdot \left( {1 + \frac{{\left[ I \right]}}{{K_{i} }}} \right) + \left[ S \right] \cdot \left( {1 + \frac{{\left[ I \right]}}{{\alpha \cdot K_{i} }}} \right)} \right\}}}} \right. \kern-\nulldelimiterspace} {\left\{ {K_{{\text{m}}} \cdot \left( {1 + \frac{{\left[ I \right]}}{{K_{i} }}} \right) + \left[ S \right] \cdot \left( {1 + \frac{{\left[ I \right]}}{{\alpha \cdot K_{i} }}} \right)} \right\}}}$, with all the measurements and estimated ${V}_{\text{max}}$ and ${K}_{\mathrm{m}}$ values. If the lower and upper limits of the 95% confidence interval (CI) of $\alpha$ were larger than 1 and 10, respectively, the mode of inhibition (MOI) was predicted to be “competitive”; if the lower and upper limits of the 95% CI of $\alpha$ were smaller than 1 and between 1 and 10, respectively, the MOI was predicted to be “Noncompetitive”; and the MOI for all other conditions was predicted to be “Mixed”. The nls() function under the R environment (v.3.5.2; The R Foundation, Vienna, Austria) was used to perform the non-linear regression model fitting, including the estimations of the 95% confidence intervals of all the estimated parameters.

### FDA pregnancy category

We searched for the labels of all the clinically approved drugs from the U.S. National Library of Medicine (NLM) DailyMed website (https://dailymed.nlm.nih.gov/dailymed/).

## Supplementary Information

Below is the link to the electronic supplementary material.

Supplementary file1 (PDF 169 KB)

Supplementary file2 (XLSX 70 KB)

Supplementary file3 (XLSX 14 KB)
