# Supplementary material for: Structure-based virtual screening of CYP1A1 inhibitors: towards rapid tier-one assessment of potential developmental toxicants
Source: Arch Toxicol. 2021 Jun 28;95(9):3031–48. doi: 10.1007/s00204-021-03111-2 (PMC8380238; doi:10.1007/s00204-021-03111-2)
Supplement: Supplementary file 1 — Supplementary file1 (PDF 169 KB) [file 204_2021_3111_MOESM1_ESM.pdf]

Structure-based virtual screening of CYP1A1 inhibitors: towards rapid tier-one assessment of potential developmental toxicants

Janice Jia Ni Goh[a, ‡], Julian Behn[a, ‡], Cheng-Shoong Chong[a, b], Guorui Zhong[a],
Sebastian Maurer-Stroh[a, b, c], Hao Fan[a, d, e *], Lit-Hsin Loo[a, f *]

[a] Innovations in Food and Chemical Safety Programme and Bioinformatics Institute, Agency for Science, Technology, and Research, Singapore
[b] Integrative Sciences and Engineering Programme, NUS Graduate School, National University of Singapore, Singapore
[c] Department of Biological Sciences, National University of Singapore, Singapore
[d] Synthetic Biology Translational Research Program, School of Medicine, National University of Singapore, Singapore
[e] Duke-NUS Medical School, Singapore
[f] Department of Pharmacology, Yong Loo Lin School of Medicine, National University of Singapore, Singapore

‡ Both authors contributed equally to this work

* Correspondence should be addressed to HF: fanh@bii.a-star.edu.sg, and LHL: loolh@bii.a-star.edu.sg

Supporting Information

Table S1. Residues from the CYP1A1 orthosteric site 2
Table S2. Residues from the CYP1A1 allosteric site 3
Table S3. Summary of reported POR-binding residues in the CYP1A family 4
References 5

Table S1. Residues from the CYP1A1 orthosteric site

Residues within 6 Å distance to at least one non-hydrogen atom of the co-crystallized ligand (α-naphthoflavone) in CYP1A1 structure 4I8V

115-ILE
116-SER
120-SER
122-SER
123-PHE
222-ASN
224-PHE
225-GLY
254-LEU
255-ASN
258-PHE
312-LEU
313-ASP
314-LEU
316-GLY
317-ALA
318-GLY
319-PHE
320-ASP
321-THR
386-ILE
496-LEU
497-THR

Table S2. Residues from the CYP1A1 allosteric site

Residues within 6 Å distance to at least one non-hydrogen atom of the docked compound Ketoconazole in CYP1A1 structure 4I8V.

89-LEU
93-ARG
97-VAL
373-LEU
376-PHE
411-GLN
441-LYS
444-SER
445-GLU
446-LYS
447-VAL
448-ILE
449-ILE
450-PHE
451-GLY
452-MET
453-GLY
454-LYS
456-LYS
460-GLU
464-ARG

Table S3. Summary of reported POR-binding residues in the CYP1A family

CYP1A enzyme	Organism	POR binding residues	UniProt accession ID (canonical)	Corresponding positions on human CYP1A1 (P04798)	References
**CYP1A1**	*Rattus norvegicus*	K97	P00185	**R93**	1
		K271		K267	1-2
		K279		K275	1-2
		C293		C289	3
		K407		K403	1
**CYP1A2**	*Rattus norvegicus*	K94	P04799	**R93**	4
		K99		R98	4-5
		K105		K104	4
		R135		R134	4
		R136		R135	4
		R137		R136	4
		K440		**K441**	4
		K453		**K454**	4-6
		R455		**K456**	4-5
		K463		**R464**	4
**CYP1A2**	*Oryctolagus cuniculus*	Y244	P00187	Y242	7
		Y272		Y270	7

Residues in **bold** overlap with the predicted allosteric binding site for ketoconazole

References

1. Shen, S.; Strobel, H. W., The role of cytochrome P450 lysine residues in the interaction between cytochrome P450IA1 and NADPH-cytochrome P450 reductase. Arch Biochem Biophys 1992, 294 (1), 83-90.
2. Cvrk, T.; Strobel, H. W., Role of LYS271 and LYS279 residues in the interaction of cytochrome P4501A1 with NADPH-cytochrome P450 reductase. Arch Biochem Biophys 2001, 385 (2), 290-300.
3. Parkinson, A.; Thomas, P. E.; Ryan, D. E.; Gorsky, L. D.; Shively, J. E.; Sayer, J. M.; Jerina, D. M.; Levin, W., Mechanism of inactivation of rat liver microsomal cytochrome P-450c by 2-bromo-4'-nitroacetophenone. Journal of Biological Chemistry 1986, 261 (25), 11487-11495.
4. Shimizu, T.; Tateishi, T.; Hatano, M.; Fujii-Kuriyama, Y., Probing the role of lysines and arginines in the catalytic function of cytochrome P450d by site-directed mutagenesis. Interaction with NADPH-cytochrome P450 reductase. J Biol Chem 1991, 266 (6), 3372-5.
5. Mayuzumi, H.; Sambongi, C.; Hiroya, K.; Shimizu, T.; Tateishi, T.; Hatano, M., Effect of mutations of ionic amino acids of cytochrome P450 1A2 on catalytic activities toward 7-ethoxycoumarin and methanol. Biochemistry 1993, 32 (21), 5622-5628.
6. Furuya, H.; Shimizu, T.; Hirano, K.; Hatano, M.; Fujii-Kuriyama, Y.; Raag, R.; Poulos, T. L., Site-directed mutageneses of rat liver cytochrome P-450d: catalytic activities toward benzphetamine and 7-ethoxycoumarin. Biochemistry 1989, 28 (17), 6848-6857.
7. Janig, G. R.; Kraft, R.; Blanck, J.; Ristau, O.; Rabe, H.; Ruckpaul, K., Chemical modification of cytochrome P-450 LM4. Identification of functionally linked tyrosine residues. Biochim Biophys Acta 1987, 916 (3), 512-23.
